# *In silico* analysis suggests repurposing of ibuprofen for prevention and treatment of EBOLA virus disease

**DOI:** 10.12688/f1000research.6436.1

**Published:** 2015-05-01

**Authors:** Veljko Veljkovic, Marco Goeijenbier, Sanja Glisic, Nevena Veljkovic, Vladimir R. Perovic, Milan Sencanski, Donald R. Branch, Slobodan Paessler

**Affiliations:** 1 Center for Multidisciplinary Research, Institute of Nuclear Sciences Vinca, University of Belgrade, Mihajla Petrovica 12-14, 11001 Belgrade, Serbia; 2 Department of Viroscience, Erasmus MC, Rotterdam, Netherlands; 3 Canadian Blood Services, Center for Innovation, 67 College Street, Toronto, M5G 2M1, Canada; 4 Department of Pathology, Galveston National Laboratory, University of Texas Medical Branch, 301 University Boulevard, Galveston, TX, USA

**Keywords:** drug space, NSAID, molecular libraries

## Abstract

The large 2014/2015 Ebola virus outbreak in West Africa points out the urgent need to develop new preventive and therapeutic approaches that are effective against Ebola viruses and  can be rapidly utilized. Recently, a simple theoretical criterion for the virtual screening of molecular libraries for candidate inhibitors of Ebola virus infection was proposed. Using this method the ‘drug space’ was screened and 267 approved and 382 experimental drugs as candidates for treatment of the Ebola virus disease (EVD) have been selected. Detailed analysis of these drugs revealed the non-steroidal anti-inflammatory drug ibuprofen as an inexpensive, widely accessible and minimally toxic candidate for prevention and treatment of EVD. Furthermore, the molecular mechanism underlying this possible protective effect of ibuprofen against EVD is suggested in this article.

## Introduction

The recent Ebola virus outbreak in West Africa caused (as of April 8, 2015) a total of 25,556 confirmed cases, including 10,587 deaths ( World Health Organization, Ebola data and statistics). Although reports of new, confirmed cases of Ebola seemed to decrease in April of 2015 to approximately 30 cases per week, medical and aid organizations are warning that the current crisis is not over. Furthermore, there remains a risk for future epidemics. Therefore, there is an urgent need to develop new preventive and therapeutic approaches that can be rapidly utilized. New drugs for the treatment of EVD should be safe, efficacious, easy to manufacture and inexpensive in order to be successfully deployed in African countries. In contrast to the significant progress recently achieved in development of an effective Ebola virus vaccine ^1^, therapeutic options are still limited ^2^. The main obstacle represents identification of an appropriate therapeutic target, which has been largely hampered by the time and money-consuming development of new drugs, especially for neglected diseases. Furthermore, the registration of newly identified drugs, like favipiravir, for potential usage in EVD patients takes a relatively long period of time, even though it has been shown to be effective in non-human primate models. In order to avoid these obstacles, several approaches for repurposing of approved drugs for the treatment of EVD patients have been proposed in literature ^3– 7^.

Recently, we proposed a relatively simple theoretical criterion for the fast virtual screening of molecular libraries for candidate inhibitors of Ebola virus infection ^8^. Using this criterion, which is based on calculation of the average quasi-valence number (AQVN) and the electron-ion interaction potential (EIIP) - parameters determining long-range interaction between biological molecules ^9– 13^ - we selected 267 approved and 382 experimental drugs as candidates for treatment of EVD. Further detailed analysis of these drugs, including molecular docking, revealed ibuprofen as an inexpensive, widely accessible and minimally toxic candidate for potential prevention and treatment of EVD. The molecular mechanism underlying the possible protective effect of ibuprofen against EVD is suggested.

## Material and methods

### AQVN and EIIP molecular descriptors

Recently, we proposed a theoretical criterion for selection of candidate inhibitors of Ebola virus infection ^8^. This criterion is based on the calculation of EIIP and AQVN which are determined by following equations:

$$Z^{*}=\frac{1}{N}\sum_{i=1}^{m}n_{i}Z_{i}\;(1)$$

where:

*i* - Type of the chemical element
Z _*i*_ - Valence of the *i*-th chemical element
n _*i*_ - Number of the *i*-th chemical element atoms in the compound
m - Number of types of chemical elements in the compond
N - Total number of atoms

$$EIIP=0.25\frac{Z^{*}\sin(1.04\;\pi \;Z^{*})}{2\pi }\;(2)$$

The EIIP values calculated according to the equation (2) are in Rydbergs (Ry = 13.6 eV).

The AQVN and EIIP molecular descriptors determine the long-distance (>5Å) intermolecular interactions in biological systems ^14^. This approach showed that molecules which potentially block Ebola virus infection are placed within AQVN range (2.3–2.7) and EIIP range (0.829–0.954 Ry), respectively. Using this theoretical criterion the drug library encompassing 267 approved drugs selected as candidate inhibitors of the Ebola virus infection (Candidate Ebola Drugs Database, CEDD) was established ^15^. This drug library was used for selection of an approved drug, which represents the optimal candidate for prevention and treatment of EVD.

### GP1 receptor modelling

The modelling of glycoprotein GP1 from Ebola virus was previously described in 16.

### Ligand optimization

Ligands were built in VEGA ZZ ^17^, protonated according to physiological conditions and optimized on semi empirical PM6 level of theory using MOPAC 2009.

### Molecular docking

Ligand and receptor were prepared in VEGA ZZ ^17^. The docking was carried out with Autodock Vina (version 1.1.2.) ^18^. In both cases the whole receptor conformational space was searched, using grid boxes with dimensions 60×60×60 and 30×30×30Å ^3^. The docking was carried out with weighting of hydrophilic interactions, and the corresponding parameter value in the docking configuration was set to -1.20 (compared to default weight: hydrogen = -0.587439). The exhaustiveness was set to 250. The conformations with lowest binding energy values were chosen. The calculations were carried out on the PARADOX Cluster computer.

## Results and discussion

We screened the CEDD library ^15^ for optimal candidates for prevention and treatment of EVD. To achieve this end, we used the following criteria: a) high efficacy of binding of drug to the Ebola virus glycoprotein GP1; b) low toxicity and accessibility (nonprescription drugs); and c) low cost. By mining of CEDD using these criteria, we selected ibuprofen as the best candidate. Ibuprofen is a nonsteroidal anti-inflammatory drug (NSAID). Its mode of action, as for other NSAIDs, is not completely understood, but may be related to prostaglandin synthetase inhibition. It achieves this effect on prostaglandin synthesis by inhibiting cyclooxygenase (COX I and II isotypes), an enzyme that is present in at least one isoform most of the tissues of the body and which is responsible for production of not only prostaglandins but also prostacyclins and tromboxane.

Generally, ibuprofen is minimally toxic drug. A meta-analysis of eight placebo-controlled studies showed that application of non-prescription doses of ibuprofen (800–1200 mg/day) over 10 days caused no significant adverse events ^19^. Similar results were obtained in a prospective study of ibuprofen use in healthy volunteers taking the maximum permitted, non-prescription dose of ibuprofen (1200 mg/day) for 10 days ^20^. It was demonstrated that administration of ibuprofen in children is safe. A randomized, community-based study on the safety of ibuprofen in febrile children aged 2 years old (5 or 10 mg/kg) showed that the risk of hospitalization for gastrointestinal bleeding associated with this drug was 17 per 100,000 ^21^. It was also shown that fecal blood loss associated with application of prescription doses of ibuprofen (2400 mg/day) did not exceed the normal range ^22^. Even in the case of post-surgical usage for pain control in children undergoing tonsillectomy, ibuprofen administration is considered to be safe and does not increase risk of hemorrhages ^23^. Accordingly, we believe that it would be crucial to better understand the potential risk and benefits of ibuprofen usage in experimental models of EVD. Taken together, these data indicate that ibuprofen could be safely used in nonprescription doses in EVD patients, with potential antiviral effects as well as to alleviate the symptoms.

**Figure 1.**
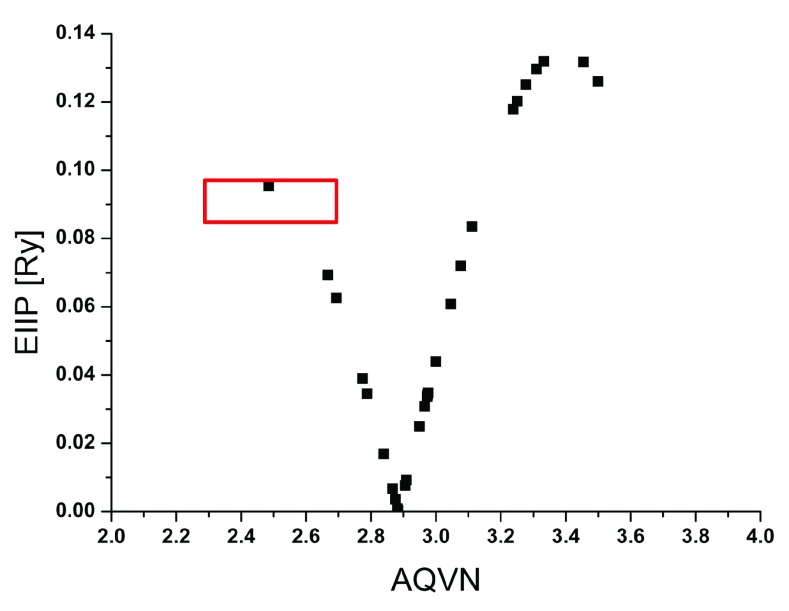
Distribution of nonsteroidal anti-inflamatory drugs in the AQVN/EIIP space. Domain of inhibitors of Ebola virus infection ^8^ is marked in red.

To assess specificity of ibuprofen as a potential inhibitor of the Ebola virus infection, distribution of all approved NSAIDs in AQVN/EIIP space was analyzed. Results presented in ( Table 1 and Figure 1) show that only ibuprofen and its isomer dexibiprofen are located within the domain of the AQVN/EIIP space. Interestingly, most of the experimentally verified inhibitors of the Ebola virus infection are located in the same space (e.g. chloroquine, amodiaquine, brincidofovir, etc) ^8^. This suggests that ibuprofen and dexibuprofen are the only drugs from the NSAID group that potentially have anti-Ebolavirus effects, which should be tested both *in vitro* and *in vivo*.

**Table 1.** AQVN and EIIP molecular descriptors of nonsteroidal anti-inflammatory drugs.

NSAID	Formula	AQVN	EIIP [Ry]
Aspirin	C9H8O4	3.238	0.1179
Diflunisal	C13H8F2O3	3.077	0.0720
Salicylic acid	C7H6O3	3.250	0.1202
Salsalate	C14H10O5	3.310	0.1296
**Ibuprofen**	**C13H18O2**	**2.485**	**0.0954**
**Dexibuprofen**	**C13H18O2**	**2.485**	**0.0954**
Naproxen	C14H14O3	2.839	0.0169
Fenoprofen	C15H14O3	2.875	0.0036
Ketoprofen	C16H14O3	2.909	0.0092
Dexketoprofen	C16H14O3	2.909	0.0925
Flurbiprofen	C15H13FO2	2.774	0.0390
Oxaprozin	C18H15NO3	2.973	0.0337
Loxoprofen	C15H18O3	2.667	0.0693
Indomethacin	C19H16ClNO4	2.976	0.0347
Tolmetin	C15H15NO3	2.882	0.0008
Sulindac	C20H17FO3S	2.905	0.0076
Etodolac	C17H21NO3	2.667	0.0693
Ketorolac	C15H13NO3	3.000	0.0439
Diclofenac	C14H11Cl2NO2	2.867	0.0067
Aceclofenac	C16H13Cl2NO4	3.000	0.0439
Nabumetone	C15H16O2	2.667	0.0693
Piroxicam	C15H13N3O4S	3.277	0.1251
Meloxicam	C14H13N3O4S2	3.333	0.1319
Tenoxicam	C13H11N3O4S2	3.454	0.1317
Droxicam	C16H11N3O5S	3.500	0.1260
Lornoxicam	C13H10ClN3O4S2	3.454	0.1317
Isoxicam	C14H13N3O5S	3.333	0.1319
Mefenamic acid	C15H15NO2	2.788	0.0345
Meclofenamic acid	C14H11Cl2NO2	2.867	0.0067
Flufenamic acid	C14H10F3NO2	2.867	0.0067
Tolfenamic acid	C14H12ClNO2	2.867	0.0067
Celecoxib	C17H14F3N3O2S	2.950	0.0249
Rofecoxib	C17H14O4S	3.111	0.0835
Valdecoxib	C16H14N2O3S	3.111	0.0835
Parecoxib	C19H18N2O4S	3.046	0.0608
Lumiracoxib	C15H13ClFNO2	2.788	0.0345
Etoricoxib	C18H15ClN2O2S	2.974	0.0342
Firocoxib	C17H20O5S	2.884	0.0003
Nimesulide	C13H12N2O5S	3.333	0.1319
Clonixin	C13H11ClN2O2	2.966	0.0308
Licofelone	C23H22ClNO2	2.694	0.0626

**Figure 2.**
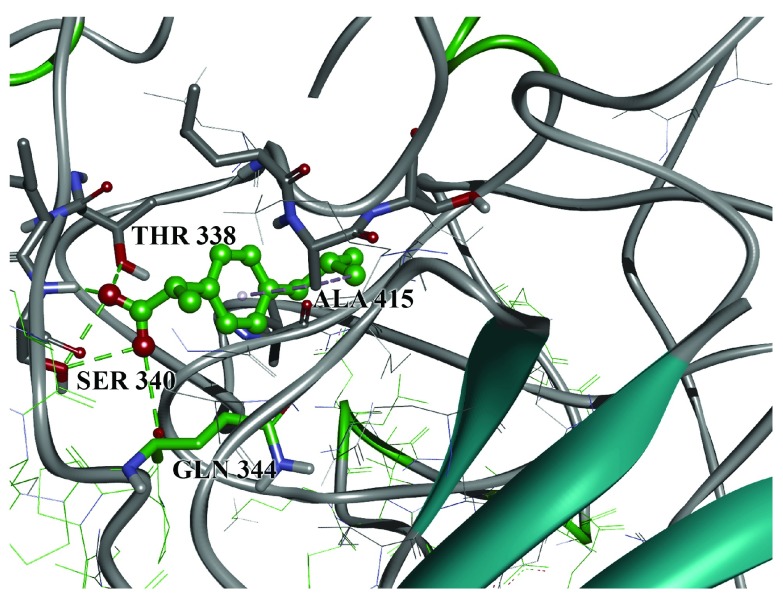
Ibuprofen docked to GP1 with marked amino-acid residues. Green dotted lines: hydrogen bonds; grey: hydrophobic interactions.

A previous *in silico* study suggested that Elastin Microfibril Interface Located Proteins (EMILINs) are involved in interaction between GP1 and endothelial extracellular matrix (ECM) ^16^. Docking of ibuprofen to the GP1 model gave conformations which bound to EMILINs binding domain on GP1 ^16^. The binding site of ibuprofen on GP1 spans the edge of this region and consists of Thr 338, Ser 340, Gln 344 and Ala 415 ^16^. The intermolecular interactions between ibuprofen and binding site amino-acids include hydrogen bonds of carboxyl group with Thr 338, Ser 340 and Gln 334. Additional stabilization is provided through aromatic and hydrophobic interaction with Ala 415. This binding site is placed between two loops, which provide the possibility of stabilizing a particular conformation, and therefore possibly blocking receptors. The appropriately high binding energy of -9.0 kcal/mol favors this assumption. The binding conformation is presented in Figure 2. At this stage it can be hypothesized that ibuprofen prevents interaction between Ebola virus and ECM by blocking the interaction between GP1 and EMILIN. There are some literature data that support our current hypothesis. EMILIN-1 is a glycoprotein expressed in the vascular tree that binds to the TGF-β1 precursor and prevents its processing by cellular protease furin ^24^. It was shown that Emilin-1 knockout mice display increased TGF-β1 signaling in the walls of their blood vessels, leading to peripheral vasoconstriction and arterial hypertension ^25^. These matrix-dependent changes in the vascular hemodynamics caused by TGF-β1 and EMILIN-1 are important because they ultimately affect the cardiovascular morbidity and mortality rates. Recently, it was shown that activation of the TGF-β1 signaling pathway by Ebola virus plays an important role in pathogenesis of EVD ^26^. These findings suggest the possibility that binding of GP1 to EMILIN-1 prevents its interaction with TGF-β1, which results in activation of TGF-β1 signaling pathway. Binding of ibuprofen to GP1 could prevent GP1/EMILIN-1 interaction allowing EMILIN-1 to keep control of TGF-β1 signaling pathway.

In conclusion, presented results should encourage further investigation of ibuprofen and ibuprofen-inspired drugs as inexpensive, low-toxic and wide-accessible candidates for prevention and its usage in the treatment of EVD.

## Data availability

F1000Research: Dataset 2. Approved and experimental drugs selected as candidate for treatment of EVD, 10.5256/f1000research.6110.d42877 ^15^
