# Supplementary material for: In silico analysis suggests repurposing of ibuprofen for prevention and treatment of EBOLA virus disease
Source: F1000Res. 2015 May 1;4:104. [Version 1] doi: 10.12688/f1000research.6436.1 (PMC4482208; doi:10.12688/f1000research.6436.1)
Supplement: Peer review history

# Peer review: Referee response for version 1

**DOI:** 10.5256/f1000research.6904.r8796

**Published:** 2015-06-23

**Authors:** Ayub Darji

**Affiliations:** 1 Centre for Research into Animal Health (CReSA), Autonomous University of Barcelona, Barcelona, Spain

Current manuscript by Veljkovic *et al., *deals with the identification of inhibitors against Ebola virus infection. Authors took advantage of existing approved and experimental drugs and, based on *in silico *analysis, proposed ibuprofen as a potential candidate for prevention and treartment of Ebola virus infection. EIIP and AQVN calculation, previously described by authors, was employed to define ibuprofen as the best candidate against Ebola virus infection.

The manuscript is adequately presented and can be considered as acceptable for indexation.

Minor points:

Ebola virus infection is initiated by interaction between the virus glycoprotein GP1 and its cognete receptor(s). Putative receptor binding domain of GP1, key residues involved in GP1 protein folding or structure and a putative receptor binding pocket has been proposed and mapped to the N-terminal 150 amino acids of GP1 (33-185 residues).

Authors should include in the discussion as how interaction of ibuprofen with GP1 (residues, Thr 338, Ser 340, Gln 344 and Ala 415) will influence the receptor binding domain.

I have read this submission. I believe that I have an appropriate level of expertise to confirm that it is of an acceptable scientific standard.

# Peer review: Referee response for version 1

**DOI:** 10.5256/f1000research.6904.r8913

**Published:** 2015-06-05

**Authors:** Mattia Mori

**Affiliations:** 1 Center for Life Nano Science@Sapienza, Istituto Italiano di Tecnologia, Rome, Italy

In the work of Velijkovic *et al.*, *in silico *methods were used with the aim of identifying inhibitors of Ebola virus infection. Particularly, authors focused their investigation on approved and experimental drugs and proposed ibuprofen as a candidate for prevention and treatment of Ebola virus. It is well known that Ebola virus represents an international health emergency and that there is an urgent need for therapeutics and prophylactic drugs.

In the present manuscript, authors applied a previously described and well-established criterion, based on EIIP and AQVN calculation, to select ibuprofen as the best candidate for Ebola virus disease treatment. Moreover, a mechanism of action consisting on GP1 inhibition has been proposed.

It is the opinion of the present referee that some minor issues should be fixed before the manuscript may be considered as acceptable for indexation:

1. Since molecular docking of ibuprofen was performed against the putative target GP1, it would be important to report the predicted binding energy (in kcal/mol) or score, and to compare this value with estimated or experimental affinity of reference GP1 ligands. Moreover, in a repurposing perspective, it would be interesting if authors could comment on the approximated dosage of ibuprofen they expect for providing anti-Ebola activity.
2. Ibuprofen has a serum half-life of 1.8 to 2.0 hours. Did authors consider this aspect when claiming that ibuprofen could be also administered to patients to prevent Ebola virus disease.
3. Liver and gastrointestinal tissues are among the most injured by Ebola virus infection. Moreover, it is well known that ibuprofen can cause serious gastrointestinal toxicity and should be used with caution in person with coagulation defects. Did authors considered ibuprofen side effects in the context of Ebola infection treatment? Following the question 1., a comment on the expected dosage would help to clarify most of these issues.
4. It is not clear how ibuprofen can alleviate the symptoms of Ebola virus disease, also considering the proposed mechanism of action.

I have read this submission. I believe that I have an appropriate level of expertise to confirm that it is of an acceptable scientific standard.
